# Antibacterial mass drug administration for child mortality reduction: Opportunities, concerns, and possible next steps

**DOI:** 10.1371/journal.pntd.0007315

**Published:** 2019-05-23

**Authors:** Isaac I. Bogoch, Jürg Utzinger, Nathan C. Lo, Jason R. Andrews

**Affiliations:** 1 Divisions of General Internal Medicine and Infectious Diseases, Toronto General Hospital, University Health Network, Toronto, Canada; 2 Department of Medicine, University of Toronto, Toronto, Canada; 3 Swiss Tropical and Public Health Institute, Basel, Switzerland; 4 University of Basel, Basel, Switzerland; 5 Division of Infectious Diseases and Geographic Medicine, Stanford University School of Medicine, Stanford, California, United States of America; 6 Division of Epidemiology, Stanford University School of Medicine, Stanford, California, United States of America; The Hospital for Sick Children, CANADA

Mass drug administration (MDA) campaigns are designed to empirically treat at-risk populations for neglected tropical diseases caused by infections with bacteria (e.g., trachoma) and parasites (e.g., schistosomiasis and soil-transmitted helminthiasis) in a defined geographic region. Decisions to initiate treatment in communities are often made based on predefined prevalence thresholds, but once treatment is undertaken, it is typically provided to all individuals of certain age groups (e.g., school-age children or all community members aged 5 years and above) without individual diagnosis. Such campaigns are a hallmark of neglected tropical disease control and elimination in low- and middle-income countries (LMICs) [1, 2]. Recent success stories include considerable reductions in the global incidence of lymphatic filariasis [3], onchocerciasis [4], and trachoma [5] through MDA programs, as well as reduction in the prevalence and intensity of soil-transmitted helminth infection and schistosomiasis [6, 7].

A recent cluster-randomized trial conducted in Malawi, Niger, and Tanzania revealed that twice-annual MDA with azithromycin for children aged 1–59 months reduced all-cause mortality by approximately 14% [8]. A major distinction between this study and prior MDA efforts is that the treatment was not targeted to particular pathogens but rather was trialed on the basis of beneficial, unintended effects that were seen in the context of earlier azithromycin MDA programs for trachoma [9, 10]. Although the specific mechanisms for mortality reduction have yet to be elucidated, it is hypothesized that azithromycin may have prevented deaths from respiratory infection, infectious diarrhea, and malaria in treated children and even perhaps in close contacts of treated children in the community (similar to herd immunity seen with vaccination campaigns). Of note, pneumonia, diarrhea, and malaria are leading causes of infection-related death in pediatric populations from LMICs [11]. Hence, this finding may be seen as an obvious success story; lives can be saved with a simple and relatively inexpensive MDA intervention. Interestingly, a 2-year follow-up of the multicountry study [8] focusing on Tanzanian study sites did not demonstrate mortality reduction or differences in rates of diarrhea, fever, anemia, or cough between azithromycin or placebo treatment arms [12].

Health policy makers are now reviewing evidence for azithromycin MDA to consider whether such programs should be scaled up in settings with high childhood mortality. Although we agree with the urgency of finding effective solutions to address this public health issue, in our view, utmost caution is needed before scaling up MDA with antibacterial agents beyond existing trachoma control and elimination programs. We are convinced about the crucially important role of anthelmintic MDA programs for large-scale morbidity control of parasitic worm (helminth) infections; however, the use of antibacterial agents poses considerably greater threats both within communities where MDA is performed and more globally.

A major concern when administering any antimicrobial agent on a mass scale, be it an antibacterial or anthelmintic drug, is the development and subsequent spread of drug resistance. As articulated by the O’Neill commission in 2016, antimicrobial resistance (AMR) is one of the biggest global health threats of our time [13]. The number of global deaths related to AMR is currently unknown because of inadequate surveillance, but contemporary projections of several hundred thousand deaths per year are gaining recognition, and these numbers are expected to rise [14]. Global efforts to reduce antimicrobial use are slow, but large-scale stewardship campaigns are taking shape with plans to both reduce consumption and facilitate the development of new antibacterial compounds [15].

Bacteria and helminths have very different mechanisms and time courses for developing resistance. Indeed, resistance appears to emerge more readily and rapidly in bacteria, which have a shorter reproductive time and can share resistance genes horizontally, facilitating their spread [16]. AMR was already detected in the 1940s, shortly after the discovery and first use of penicillin, and has developed across all classes of antibiotics [17]. As early as 1995, *Streptococcus pneumoniae* resistance was documented in children treated with a single dose of azithromycin as part of trachoma control programs, with 1.3% of swabs demonstrating resistance before MDA and 21.3% of samples demonstrating resistance up to 2 months following treatment [18]. A more recent prospective study from a trachoma control program in Niger demonstrated significantly more macrolide-resistant *S*. *pneumoniae* strains in children from communities treated with azithromycin twice per year (60%) compared with communities treated once per year (40%) [19]. Although other studies also documented an increase in resistant *S*. *pneumoniae* strains after azithromycin MDA [20], some studies did not make this observation [21, 22]. It appears that communities with a higher baseline burden of azithromycin-resistant *S*. *pneumoniae* are at greater risk of selecting for such resistant strains in the context of azithromycin MDA [23].

Although much of the focus in macrolide MDA is on resistant *S*. *pneumoniae*, enteric infections present another major threat [24, 25]. Many gastrointestinal pathogens are now resistant to commonly used antibacterial agents after years of unselective use, and effective treatment options are increasingly limited. Azithromycin is now one of the first-line oral drug choices for treatment of shigellosis, enteric fever (caused by *Salmonella* Typhi and Paratyphi A), and invasive nontyphoidal salmonelloses, all of which have seen widespread fluoroquinolone nonsusceptibility following years of overuse and misuse [26]. At present, azithromycin is the only oral treatment option available for an emerging “extensively drug resistant” (XDR) *Salmonella* Typhi strain [27], which is resistant to third-generation cephalosporins [28]. There are also many other diarrhea-causing Enterobacteriaceae with emerging azithromycin resistance genes [16], and we can only expect more macrolide resistance to develop in the near future in these organisms in the face of heightened indiscriminate use.

It is conceivable that the mortality benefits of azithromycin MDA [8] will have only a narrow window before the prevalence of AMR organisms reaches levels that negate these benefits in their intended communities. Moreover, recent experience has demonstrated that resistant bacterial infections, including *Salmonella* and *Shigella*, may spread far beyond their community of origin within years [26, 29] and negatively impact communities distant from where a targeted MDA program is offered (Table 1). When macrolide AMR prevalence rises, these drugs are no longer recommended as a first-line empiric treatment for common infections like community-acquired pneumonia or infectious diarrheal illness because of the possibility of treatment failure [30]. With nonselective overuse of azithromycin, a previously effective, relatively inexpensive, widely available, and well-tolerated oral therapy will no longer be useful for areas that are most in need, and there is a shrinking list of alternative antibiotics with these same attributes.

**Table 1:** Summary of evidence for MDA campaigns targeting helminth infections versus nonselective targets of antibacterial agents.

Evidence	Helminth	Bacteria
Risk of developing AMR	Limited in human populations	Rapid emergence in human populations
Risk for subsequent AMR transmission	Limited to ecological niches of helminth	Local and global transmission over short time periods
“Off-target” effects of treatment	Relatively limited to very few helminths in the targeted MDA region	Many antibacterial agents (e.g., azithromycin) are not selective and have broad activity against gram-positive and gram-negative organisms
Efficacy in reducing local transmission of disease	Considerable	Unclear which organisms are attributed to mortality reduction; uncertain efficacy in reducing local transmission
Durability	Sustained efficacy in reduced helminth transmission over years	Lack of data

Abbreviations: AMR, antimicrobial resistance; MDA, mass drug administration.

By contrast, the history of MDA campaigns against helminth infections had a different course. Indeed, MDA programs for schistosomiasis and soil-transmitted helminth infections have been in place for some two decades with praziquantel (against schistosomiasis) and albendazole or mebendazole (against soil-transmitted helminthiasis) serving as mainstays of treatment. MDA for helminth infections has also faced similar critiques. Yet, we believe that some of these critiques are misplaced, as they are largely outweighed by the benefits, as summarized in Table 1. Although the efficacy of albendazole and mebendazole varies between different species of soil-transmitted helminths [31], and certainly cure rates of schistosomiasis are lower for higher-intensity infections [32], there is no evidence for sustained anthelmintic drug resistance developing in human populations despite hundreds of millions of treatments over many years of use on a global scale. There is evidence of anthelmintic drug resistance to praziquantel developing in veterinary practice and sporadic evidence in helminths infecting humans [33]; however, clinically relevant anthelmintic resistance has not emerged on a mass scale considering the enormous breadth of schistosomiasis MDA campaigns [34]. Additionally, because of complex life cycles and ecological niches of helminths, their geographic range is far more limited, and resistance—if emergent—is unlikely to spread as swiftly as it may in bacterial infections. Numerous examples of rapid global dissemination of bacterial resistance genes underscore this point—for example, with the mobilized colistin resistance gene (*mcr-1*) [35] and the New Delhi metallo-β-lactamase-1 gene (*NDM-1*) [36] developing locally but spreading globally over a short time, both of which portend resistance to most classes of antibacterial drugs.

The judicious use of all antimicrobial agents is essential to prevent the development and spread of resistance; however, much greater caution is needed when using antibacterial compared with anthelmintic agents on a mass scale. Expanding azithromycin MDA outside of trachoma programs has the potential for widespread and sustained harm secondary to AMR development and spread. More data are needed to justify the wider use of azithromycin MDA if it is to be used for survival benefit outside of trachoma control programs, and consultation with local communities, neighboring communities, and public health stakeholders should determine if the potential benefits outweigh the risks (Table 2). For helminth infections (and malaria), modeling has been used to examine tradeoffs between reductions in transmission and the rise of drug resistance [37]; for azithromycin MDA, in the absence of understanding the mechanisms of mortality reduction, such modeling is currently impossible. Moreover, it is challenging, if not impossible, to project the duration for which azithromycin MDA may have benefits before it is undermined by AMR.

**Table 2:** Suggested steps prior to implementing azithromycin MDA for mortality reduction outside of trachoma control programs.

Key step	Stakeholder(s)
**Step 1: Identify causative pathway for mortality benefit**	
• Investigate mechanism of mortality benefit and heterogeneity by study country	Academia
• Estimate duration of mortality benefit with ongoing MDA efforts	Academia
• Define AMR risk factors to consider in programmatic decision (e.g., incidence of resistant infections)	Academia
**Step 2: Estimate risk and benefit**	
• Consider overlapping risk factors (e.g., high incidence of resistance-prone bacteria)	Academia
• Discussion with public health stakeholders	Public sector; community engagement
• Model-based analysis of tradeoff between health effects and AMR risk	Academia
**Step 3: Create distinct guidelines based on epidemiology, programmatic goals, and resource constraints on MDA implementation**	
• Guidelines for geographic zone, frequency, and duration of azithromycin MDA based on defined criteria (e.g., child mortality, AMR risk)	World Health Organization; national public health organizations; community engagement

Abbreviations: AMR, antimicrobial resistance; MDA, mass drug administration.

Empirical data concerning durability of mortality reduction are needed before implementing this strategy programmatically. Until such data become available, we would recommend against azithromycin MDA in regions where AMR is common or in which azithromycin is widely used for pneumonia or gastrointestinal diseases. Instead, we suggest taking a more cautious approach and seeking other methods to reduce mortality in high-risk settings rather than expand azithromycin MDA beyond trachoma programs because of the risks of long-term harms of AMR. Should these programs expand, AMR and rigorous clinical surveillance must scale up in tandem to closely monitor for the emergence and spread of resistant pathogens, which would trigger public health responses. With the increasing global burden of highly drug-resistant bacterial infections, for which azithromycin remains a critical drug in the arsenal, the stakes are high, and the evidentiary bar for its long-term safety in widespread use should be also high.
